# Overexpression of Lectin Receptor-Like Kinase 1 in Tomato Confers Resistance to *Fusarium oxysporum* f. sp. *Radicis-Lycopersici*

**DOI:** 10.3389/fpls.2022.836269

**Published:** 2022-02-03

**Authors:** Zhi-Liang Yue, Zhe-Juan Tian, Jun-Wei Zhang, Sheng-Wei Zhang, Ya-Dong Li, Zhi-Ming Wu

**Affiliations:** ^1^Institute of Cash Crops, Hebei Academy of Agriculture and Forestry Sciences, Shijiazhuang, China; ^2^Ministry of Education Key Laboratory of Molecular and Cellular Biology, Hebei Collaboration Innovation Center for Cell Signaling and Environmental Adaptation, Hebei Key Laboratory of Molecular and Cellular Biology, College of Life Sciences, Hebei Normal University, Shijiazhuang, China

**Keywords:** *Fusarium* crown and root rot, *Fusarium oxysporum* f. sp. *radicis-lycopersici*, tomato, lectin receptor-like kinase, disease resistance

## Abstract

The disease *Fusarium* crown and root rot (FCRR), caused mainly by *Fusarium oxysporum* f. sp. *radicis-lycopersici* (*FORL*), seriously affects commercial tomato [*Solanum lycopersicum* (Sl)] yields. However, the genes that offer resistance to *FORL* are limited and the mechanism of resistance to FCRR is poorly understood. Lectin receptor-like kinases (LecRKs) play critical roles in defensive responses and immunity in many plant species; however, whether specific LecRKs are involved in the response of tomato plants to *FORL* is unclear. Here, we report that the expression of *SlLecRK1/Solyc09g011070.1* was obviously induced by the infection of *FORL*. Biochemical and cell biological data revealed that SlLecRK1 is an active kinase that is located at the cell membrane, while real-time quantitative PCR data suggested that *SlLecRK1* is mainly expressed in stems and roots. Genetic studies showed that overexpression of *SlLecRK1* significantly improved the resistance of tomato plants to *FORL* but did not cause visible changes in plant growth and development compared with wild-type control plants. RNA-Seq data suggested that the positive effects of SlLecRK1 on the resistance of tomato plants to *FORL* occur mainly by triggering the expression of *ethylene-responsive transcription factor* (*ERF*) genes. Together, our findings not only identify a new target for the development of FCRR-resistant tomato varieties, they also demonstrate a molecular mechanism linking SlLecRK1 and ERFs in regulating the immune responses of tomato plants to *FORL*.

## Introduction

Decades of research have revealed that crop succession can lead to soil-borne diseases such as *Fusarium* crown and root rot (FCRR), an emerging disease that seriously threatens tomato (*Solanum lycopersicum* L.) production in most countries (Polizzi et al., 2011; Cordero-Ramirez et al., 2013; Sepulveda-Chavera et al., 2014). FCRR can occur from the seedling stage to the fruiting stage in tomato plants. At the start of the disease, no phenotypic change is visible in the above-ground parts of the plant; however, as the disease progresses wilting and browning are observed leading to plant death. Thus, FCRR causes reduced numbers or a lack of tomatoes. FCRR is mainly caused by *Fusarium oxysporum* f. sp. *radicis-lycopersici* (*FORL*), a fungus that can survive in soil as spores for up to 6 years (Rekah et al., 2000; Gordon, 2017; Srinivas et al., 2019). The application of disinfectants and fungicides is a major approach to reduce FCRR; such chemical control is extensively used in the commercial production of tomatoes (Benhamou and Belanger, 1998; Myresiotis et al., 2012). Additionally, biological resources, including bacteria and fungi, have been used to control FCRR in tomato (Chin et al., 2000; Grauwet et al., 2005; Kavroulakis et al., 2007; Nefzi et al., 2019). However, these methods are of limited usefulness during a disease outbreak. Though the development of resistant tomato varieties would be an efficient and economical approach to the control of FCRR in tomato, our understanding of the genes conferring resistance to *FORL* is limited and the mechanisms of resistance are poorly understood (Mazzeo et al., 2014; Manzo et al., 2016).

Several studies have demonstrated that plants recognize pathogenic microorganisms *via* cell membrane proteins, termed pattern recognition receptors (PRRs; Nurnberger and Kemmerling, 2006; Monaghan and Zipfel, 2012; Park et al., 2012; Tang et al., 2017; Kong et al., 2021). Most PRRs are receptor-like kinases (RLKs) or receptor-like proteins (RLPs). Both RLKs and RLPs contain an extracellular domain that perceives extracellular signals; RLKs transduce these signals *via* their intracellular kinase domain to trigger defense-related gene expression and the synthesis of defensive chemicals, including plant hormones, reactive oxygen species, and callose to enhance plant disease resistance (Park et al., 2012; Tang et al., 2017). The roles of RLKs in response to pathogenic bacteria have been extensively studied in many plant species, and some RLKs are considered ideal candidates for molecular breeding programs (Robatzek et al., 2006; Schwessinger et al., 2015; Pizarro et al., 2018; Piazza et al., 2021). However, little is known about whether and which RLKs are involved in tomato resistance to *FORL*.

RLKs comprise a vast protein family encoded by more than 600 genes in *Arabidopsis* and 1,100 genes in rice. Based on their extracellular domains, RLKs are classified into 15 subfamilies (Vaid et al., 2013). Among these subfamilies, lectin RLKs (LecRKs) are different from RLKs in that their extracellular domain resembles that of carbohydrate-binding lectin proteins (Bouwmeester and Govers, 2009). LecRKs are thought to play vital roles in the defense of plants against various pathogens and pests (Singh and Zimmerli, 2013; Bellande et al., 2017; Wang and Bouwmeester, 2017; Sun et al., 2020), and accumulating evidence indicates that some LecRLKs act as PRRs or important regulators of plant immune responses. For instance, in *Arabidopsis*, LecRK-V.5, LecRK-IX.1, and LecRK-IX.2 contribute to resistance against *Phytophthora* spp. LecRK-VI.2 is also a positive regulator of stomatal innate immunity (Singh et al., 2012). Overexpression of LecRK-IX.2 or LecRK-IV.3 also results in enhanced resistance to *Pseudomonas syringae* pv. tomato DC3000 and *Botrytis cinerea*, respectively (Wang et al., 2015). LecRK-I.8 is required for the perception of insect egg-derived elicitors (Gouhier-Darimont et al., 2019). More importantly, recent studies have shown that LecRK-I.9/DORN1 is involved in plant immune responses by acting as a PRR for the damage-associated molecular pattern eATP; further, the extracellular domain of LecRK-I.9 binds ATP with high affinity. This binding is required for ATP-induced calcium responses, MAPK activation, and gene expression. Therefore, LecRK-I.9 probably plays a variety of roles in plant stress resistance (Choi et al., 2014; Chen et al., 2017; Wang et al., 2018).

Since LecRKs are involved in the response of plants to various pathogens, as well as in biological recognition processes involving cells and proteins (Vaid et al., 2013), the members of this subfamily also likely participate in the responses of tomato plants to *FORL*. In this study, we found that SlLecRK1, the homolog of LecRK-I.9/DORN1 (Choi et al., 2014; Chen et al., 2017; Wang et al., 2018) in tomato, was induced at the mRNA and protein levels by inoculation with *FORL*. Using genetic, molecular, and physiological strategies, we found that the overexpression of SlLecRK1 improved tomato resistance to *FORL* by triggering the expression of *ERFs*. Notably, *SlLecRK1*-overexpressing (*OE*) plants displayed no visible phenotypic change compared with wild-type (WT) control plants, demonstrating the potential of the gene in the development of FCRR-resistant tomato varieties. Thus, we conclude that *SlLecRK1* may be used in molecular breeding programs to enhance the resistance of tomato plants to *FORL*.

## Materials and Methods

### Plant Materials and Growth Conditions

The *FORL*-susceptible tomato (*S. lycopersicum*) variety *Moneymaker* was used in this study. Tomato plants were grown in a greenhouse under a 16-h light/8-h dark photoperiod at 25°C/22°C, or in a greenhouse under natural conditions (Mar-Jul in Hebei, China) for seed production. Seeds were sown on half-strength Murashige and Skoog medium containing 1.5% (w/v) sucrose and grown in a light chamber under a 16-h light/8-h dark photoperiod at 25°C/22°C.

### Fungal Isolation and Infection Assay

*FORL* was isolated from field grown, infected tomato plants, and the isolate was identified by PCR using specific primers as described previously (Validov et al., 2011). The isolate was kept in 50% glycerol at −80°C for use in our experiments. The isolate was grown in potato dextrose broth on a rotary shaker for 5 days at 23°C. The culture was then filtered through two layers of antiseptic gauze. The suspension was centrifuged at 3000 rpm for 10 min and conidia were resuspended in ddH_2_O at a spore concentration of 10^7^ CFU/ml. Roots of 10-day-old seedlings were dipped in the suspension and then transplanted at normal photoperiods for 1–3 days. For 30-day-old tomatoes, the stem bases were punctured and the suspension was poured into the soil, after which the tomatoes were allowed to keep growing.

### Plasmid Construction and the Generation of Transgenic Plants

To produce the expression constructs *SlLecRK1Pro:SlLecRK1-GFP* and *SlLecRK1Pro:SlLecRK1-Myc*, a 4,353-bp fragment of the SlLecRK1 promoter and coding sequence was amplified by PCR (*FastPfu Fly*, Cat. #AP231; TransGen Biotech) using primers flanked by a *Hind*III site (SlLecRK1Pro F) and *BamH*I site (cSlLecRK1 R); the primers are listed in Supplementary Table S1. The fragment was cloned into *pCAMBIA1300-MH* between the *Hind*III and *BamH*I sites by ligation (Cat. #M0202S; New England Biolabs) to replace the existing 35S promoter. The *SlLecRK1Pro:SlLecRK1-Myc* construct was sequenced before proceeding to tomato transformation with *Agrobacterium tumefaciens* strain EHA105. The *SlLecRK1Pro:SlLecRK1-GFP* construct was used for transient expression in *N. benthamiana* leaves.

### Fungal DNA Concentration Analysis

Plants inoculated with *FORL* were pulverized in liquid nitrogen and then mixed with 1 ml of cetyl-trimethyl-ammonium bromide. Each mixture was incubated at 95°C for 1 h and then centrifuged at 12000 × *g*. The supernatant was extracted with one volume of chloroform. The upper phase was transferred to a new Eppendorf tube and the DNA was precipitated by adding one volume of isopropanol followed by centrifugation at 12000 × *g*. The DNA pellets were washed with 70% ethanol and dried. Total DNA was dissolved in ddH_2_O and then quantified using a NanoDrop 1,000 Spectrophotometer (Thermo Fisher). To determine the fungal DNA concentration, qPCR was performed as described previously (Validov et al., 2011), using the primers OMP1049 and OMP1050, by SYBR Premix Ex Taq (Cat. #RR420A; Takara Bio Inc.) and a C1000 Touch Thermal Cycler CFX96 (Bio-Rad). A standard curve for quantification was generated by plotting the log of the concentrations (from 50 fg to 2 ng) of total DNA isolated from *FORL*; the primers used are listed in Supplementary Table S1.

### Real-Time Quantitative PCR

Total RNA was extracted from specific tissues treated (or not), as indicated, with TRIzol reagent (Cat. #15596024; Invitrogen) according to the manufacturer’s instructions. First-strand cDNA was synthesized using a PrimeScript RT Reagent Kit with Genomic DNA Eraser (Cat. #RR047A; Takara Bio Inc.) Gene expression was analyzed by qRT-PCR using SYBR Premix Ex Taq (Cat. #RR420A; Takara Bio Inc.) and a C1000 Touch Thermal Cycler CFX96 (Bio-Rad). All primers are listed in Supplementary Table S1. The relative abundance of the target transcripts was determined by the comparative threshold cycle method (Schmittgen and Livak, 2008) using *Elongation Factor 1 alpha* (*eEF1*α,Solyc06g069020) as an internal reference (Campos et al., 2019).

### Immunoblotting

Tomato seedlings were ground in liquid nitrogen as indicated and then boiled in SDS sample buffer [50 mm Tris, pH 6.8, 2 mm EDTA, 10% (w*/*v) glycerol, 2% SDS, and 6% 2-mercaptoethanol; 2 μg/μl of tissue]. A 10 μl sample from each supernatant after centrifugation at 10000 × *g* for 10 min was subjected to 10% SDS-PAGE. Separated proteins were transferred to a nitrocellulose membrane and blocked with 5% skimmed milk in phosphate-buffered saline (PBS) for 1 h. Then, the membrane was probed with the indicated primary antibodies (1:3000 in 5% skimmed milk in PBS; Mouse monoclonal anti-Myc, Cat. #M4439, Sigma-Aldrich; Mouse monoclonal anti-GFP, Cat. #G1544, Sigma-Aldrich) for at least 3 h. After three washes with wash buffer (PBS, supplemented with 0.001% Tween) for 10 min each, the membrane was probed with horseradish peroxidase (HRP)-conjugated antibodies against mouse-IgG for 1 h (Cat. #BA1051; Boster). After three washes with wash buffer (PBS, supplemented with 0.001% Tween) for 10 min each, the membrane was incubated with SuperSignal West Femto Maximum Sensitivity Substrate (Cat. #34095; Thermo Scientific) and signals were detected by CCD imaging (Zhao et al., 2019).

### Subcellular Localization

The constructs *SlLecRK1Pro:SlLecRK1-GFP* and *35SPro:GFP* were transiently expressed in *N. benthamiana* leaves, incubated with FM4-64 (Cat. #T13320; Invitrogen) for 5 min for plasma membrane staining and then visualized under laser scanning confocal microscopy (FLUOVIEW FV3000, Olympus; GFP: excitation at 488 nm and emission at 510 nm; FM4-64: excitation at 587 and emission at 610 nm).

### Preparation of Recombinant Proteins and Kinase Assay

To prepare GST-ICD and GST-mICD, the coding sequence of SlLecRK1-ICD (amino acids 310–681) was recombined into vector *pGEX-6p-1*. The resulting constructs, *pGEX-6p-1-SlLecRK1 ICD* and *pGEX-6p-1-mSlLecRK1 ICD*, were transformed into *E. coli* strain Rosetta (DE3) and the recombinant proteins were produced and purified using Glutathione Sepharose 4 Fast Flow (Cat. #17513202; Cytiva) following the manufacturer’s instructions. For GST-ICD and GST-mICD phosphorylation, 3 μg of recombinant purified proteins were incubated in kinase buffer (50 mm HEPES, pH 7.5, 10 mm MgCl_2_, 5 mm MnCl_2_, and 1 mm DTT) containing 0.2 mm ATP at 30°C for 3 h (Oh et al., 2012; Zhao et al., 2019) prior to SDS-PAGE followed by immunoblotting. Anti-phosphoserine/threonine (Cat. #PP2551; ECM) was used to detect kinase phosphorylation levels, and Anti-GST HRP (Cat. #1006–3; HuaAn Biotechnology) was used to detect GST-SlLecRK1 protein loading.

### RNA Sequencing

Total RNA was isolated from tomato seedlings using a TRIzol Kit (Invitrogen). Paired-end sequencing libraries were prepared using a TruSeq RNA Sample Preparation Kit, version 2 (Illumina) and sequenced on a HiSeq Xten machine according to the manufacturer’s protocols. FastQC^1^ was initially run to assess the overall quality of the sequenced reads. Poor-quality reads were filtered out using Sickle with the parameters pe mode; −t Sanger -q 20 -l 50.^2^ The remaining high-quality reads were mapped to the *S. lycopersicum* reference genome (ITAG4.0) using TopHat2, version 2.09 (Kim et al., 2013), with the parameters “-N 3-read- edit-dist 3-segment-mismatches 1 -p 20 -r 0 -g 20—microexon-search -b2 -D 20 -b2-R3-no-coverage-search.” Only reads showing unique alignments were retained for the following analysis. HTseq software^3^ was used to determine the read counts mapped to each of the genes. Next, genes with more than 10 reads in at least one sample were kept for differential expression analysis. Batch effects were removed from the filtered read count table using RUVseq (normalization of RNA-Seq data using a factor analysis of control genes or samples) with the residual RUVr approach. The Bioconductor package “edgeR” (Robinson et al., 2010) was used for PCA and differential expression analysis. Only genes with an FDR < 0.05 and absolute value of log2 (fold change) ≥ 0.58 (*OE-1* vs. wild type) or 1 (1 vs. 0 dpi) were considered DEGs. GO term enrichment was analyzed by singular enrichment analysis with AgriGO (Tian et al., 2017) using a hypergeometric test, with a FDR < 0.05 as the cutoff. Venn diagrams were drawn using Vennerable R.^4^

### Quantification of Protein Abundance

Protein abundance in the immunoblots was quantified by measuring the band intensity with ImageJ 1.47v.^5^ The relative protein level was obtained by normalization to the loading control.

## Results

### Solyc09g011070.1 Transcript and Protein Levels Were Increased by *FORL*

**Figure 1:**
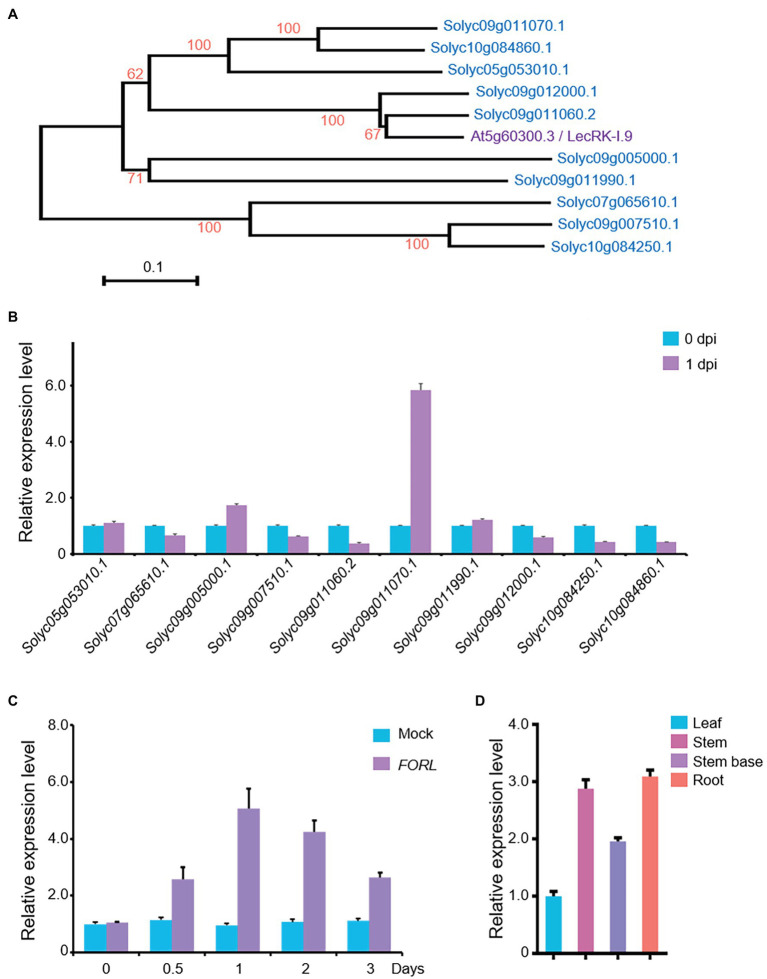
Expression of a LecRK-I.9 homolog in tomato in response to *FORL*. **(A)** A phylogenetic tree showing LecRK-I.9 and its homologs in tomato. Based on the predicted amino acid sequences using MEGA 6.0, the scale bar indicates the distance calculated from multiple alignments. **(B)** Relative expression levels of the *LecRK-I.9* homolog following *FORL* treatment in 10-day-old WT tomato seedlings as measured by qRT-PCR. Total RNA was used for analysis. *eEF1α* served as a reference. The WT values at 0 dpi were set to 1. Data are shown as the mean ± standard deviation (SD) from three biological repeats. **(C)** Induced expression of *Solyc09g011070.1* following *FORL* treatment at different time points. Total RNA was used for qRT-PCR analysis. *eEF1α* served as a reference. Ten-day-old WT tomato seedlings were treated with water (Mock) or *FORL*. Data are shown as the mean ± SD from three biological repeats. **(D)** Tissue-specific expression of *SlLecRK1* in tomato seedlings. Total RNA from the indicated tissues was used for qRT-PCR analysis. *eEF1α* served as a reference. Leaf blade values were set to 1. Data are shown as the mean ± SD from three biological repeats.

In the tomato genome, there are more than 100 genes encoding LecRKs,^6^ and 10 of them show relatively higher identity (42–47%) to LecRK-I.9/DORN1. Phylogenetic analysis also showed that the most similar protein forms a separate cluster from *Arabidopsis* LecRK-I.9/DORN1, and Solyc09g011060.2 is the most closely related gene to LecRK-I.9/DORN1 in tomato (Figure 1A). To reveal their potential roles in the *FORL* defense response in tomato, we performed qRT-PCR to assess whether the transcript levels of these *LecRKs* were affected by *FORL*. Total RNA was extracted from 10-day-old WT tomato seedlings that had been treated with *FORL* 1 day earlier. The expression of *Solyc05g053010.1* and *Solyc09g011990.1* was unchanged, while six genes were downregulated, following *FORL* treatment compared with untreated control plants. However, the transcript levels of *Solyc05g053010.1* and *Solyc09g011070.1* were obviously upregulated, and the expression level of *Solyc09g011070.1* was more than fivefold that in the control plants (Figure 1B).

Our qRT-PCR data further revealed that the mRNA expression level of *Solyc09g011070.1* was induced by *FORL* treatment from 0.5 to 3 dpi and peaked at 1 dpi, compared with mock-treated control plants (Figure 1C). Interestingly, its mRNA expression levels in the roots, stems, and stem bases of the tomato seedlings were higher than that in the leaves (Figure 1D), consistent with our findings in the tomato plant tissues exposed to *FORL* through the soil. Ultimately, we assigned *Solyc09g011070.1* the name *SlLecRK1*.

### *SlLecRK1* Encodes a LecRK

**Figure 2:**
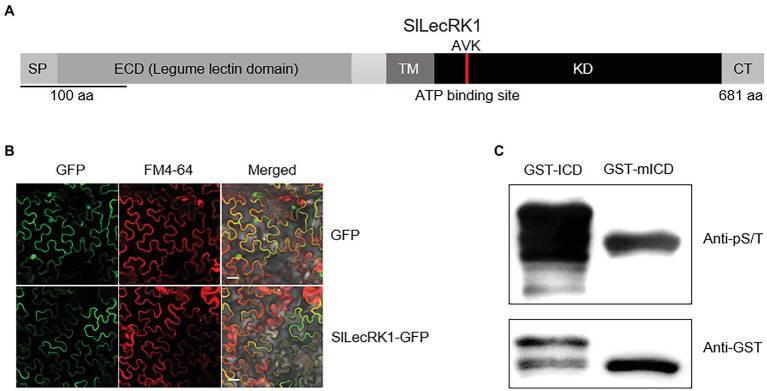
Characterization of SlLecRK1. **(A)** Diagram of the functional domains in SlLecRK1. SP, signal peptide; ECD, extracellular domain; TM, transmembrane domain; KD, kinase domain; CT, C-terminal region. The red bar in the KD marks the AVK motif (the ATP-binding site). **(B)** Confocal microscopic images of *SlLecRK1Pro:SlLecRK1-GFP* transiently expressed in *N. benthamiana* leaves. FM4-64 was used as a marker for the plasma membrane. Bars, 20 μm. **(C)** Immunoblot analysis of GST-ICD and GST-mICD recombinant proteins using antibodies specific for serine and threonine phosphorylation (pS/T). Anti-GST antibodies were used as a loading reference.

*SlLecRK1* encodes a protein of 681 amino acids, which was predicted to be a LecRK by sequence alignment. The predicted extracellular N-terminal domain contains a predicted 21-residue cleavable secretory signal peptide and a typical legume lectin domain (between residues 27 and 266). The C-terminal intracellular region is predicted to contain a protein kinase domain (between residues 343 and 613; Figure 2A).

To validate the subcellular localization of SlLecRK1, *SlLecRK1SPro:SlLecRK1-GFP* and *35SPro: GFP* (control) were transiently expressed in *Nicotiana benthamiana* leaves, respectively. Confocal observations revealed that the SlLecRK1-GFP fusion protein co-localized with the plasma membrane marker FM4-64, whereas the GFP control protein was located in the nucleus and at the cell surface (Figure 2B), indicating that SlLecRK1 localizes to the plasma membrane.

To determine whether SlLecRK1 possesses kinase activity, the conserved lysine (K^372^) in the ATP-binding region was mutated to generate SlLecRK1KD^KA^. The WT and mutated SlLecRK1 intracellular domains (ICDs) were expressed in *Escherichia coli* as glutathione S-transferase (GST) fusions, and the purified proteins were incubated with kinase buffer then detected with anti-phosphor-serine/−threonine antibodies (for details, see “Materials and Methods”). As shown in Figure 2C, the GST-ICD proteins were highly phosphorylated, whereas GST-mICD (mutated ICD) was barely phosphorylated, suggesting that SlLecRK1 has auto-phosphorylation activity and that K^372^ is required for this activity.

Together, these results suggested that SlLecRK1 is a typical LecRK in tomato.

### Overexpression of *SlLecRK1* Enhanced Tomato Resistance to FCRR

**Figure 3:**
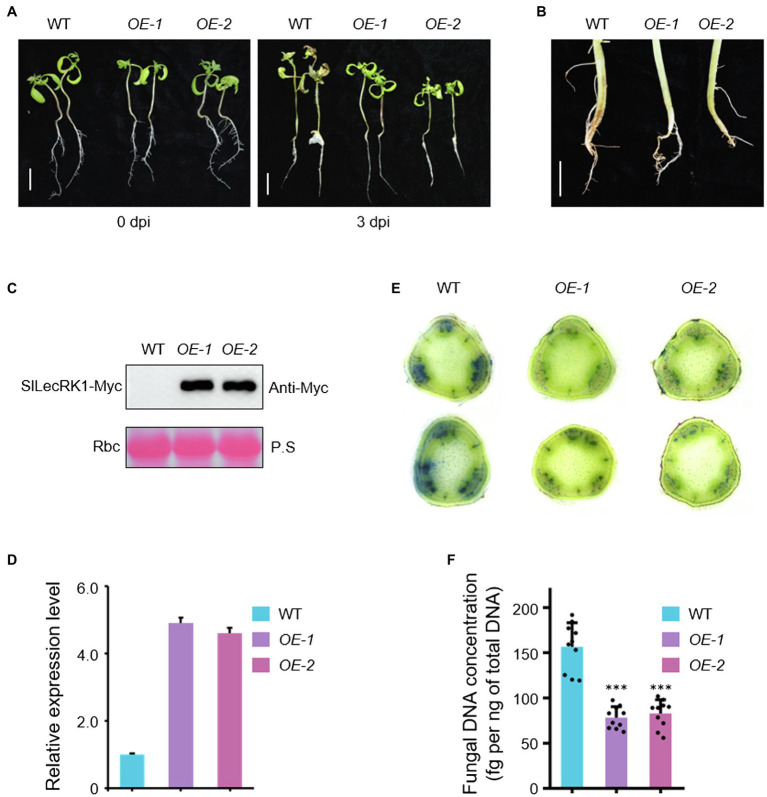
Phenotypes of *SlLecRK1*-*OE* plants in response to FCRR. **(A)** The post-*FORL* inoculation phenotypes of 10-day-old *SlLecRK1-OE* and WT tomato seedlings. Bar, 2 cm. **(B)** The post-*FORL* inoculation phenotypes of 30-day-old *SlLecRK1-OE* and WT tomato roots. Bar, 3 cm. **(C)** The SlLecRK1 protein levels in overexpression lines probed with anti-cMyc antibodies. Ponceau S (P.S.) staining of RubisCO (RBC) served as a loading reference. **(D)** The total *SlLecRK1* mRNA levels in our overexpression lines. Total RNA was used for qRT-PCR analysis. *eEF1α* served as a reference. Data are shown as the mean ± SD from three biological repeats. **(E)** Cotton blue staining indicating *FORL* abundance. Stems from WT and *OE* plants after *FORL* inoculation for 2 weeks were stained with cotton blue. Representative images of WT and *OE* plants are shown. Bars, 1 mm. **(F)** Fungal DNA concentration in WT and *OE* plants after *FORL* inoculation for 2 weeks. Different letters denote significant differences by Student’s *t*-test (^***^p < 0.001), *n* = 10.

Given that SlLecRK1 was upregulated by inoculation with *FORL*, we predicted that enhancing the expression of SlLecRK1 might be required for tomato resistance to FCRR. We therefore generated transgenic tomato plants that overexpressed SlLecRK1. *SlLecRK1Pro:SlLecRK1-Myc* was transformed into WT calli, and then the transgenic plants were validated by hygromycin resistance screening and exogenous protein detection. We selected two independent lines, *SlLecRK1-OE1* (*OE-1*) and *-OE2* (*OE-2*), which expressed SlLecRK1-Myc, for further analysis (Figures 3A–D). Ten-day-old *OE-1*, *OE-2*, and WT seedlings were inoculated with *FORL* and then transferred to fresh medium. After 3 days of cultivation, the roots, stems, and leaves of the WT plants had rotted, whereas rotted tissues were found only at the stem bases of the two *OE* lines (Figure 3A). We also injected *FORL* into the stem bases of 30-day-old plants. Two weeks later, the WT roots had turned brown and rotted from the inside out, whereas in the *OE* plants only a few lateral roots showed browning and the main roots were not obviously affected (Figure 3B).

**Figure 4:**
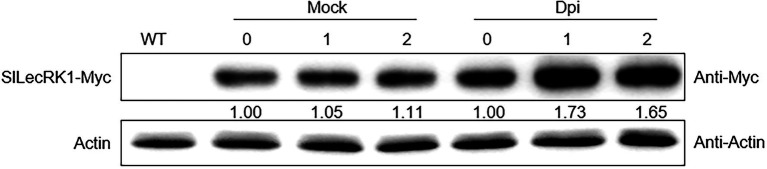
SlLecRK1 Accumulation Following Inoculation with *FORL*. Ten-day-old *SlLecRK1-OE1* seedlings were inoculated (or not) with *FORL* for the indicated number of days, after which SlLecRK1 protein was detected using anti-cMyc antibodies. The signal intensity was quantified and normalized to that of actin. The values for the day 0 samples were set to 1.

We next compared fungal accumulation at the stem in *OE* and WT plants at 2 weeks after *FORL* infestation. Cotton blue staining revealed fewer fungi in the stems of *OE-1* and *OE-2* plants compared to wild type (Figure 3E). Consistently, the concentrations of *FORL* DNA in *OE-1* and *OE-2* were about half of that in WT plants (Figure 3F). We also observed SlLecRK1 accumulation following inoculation with *FORL* (Figure 4), indicating the inhibitory effects of increased SlLecRK1 expression on fungal growth.

**Figure 5:**
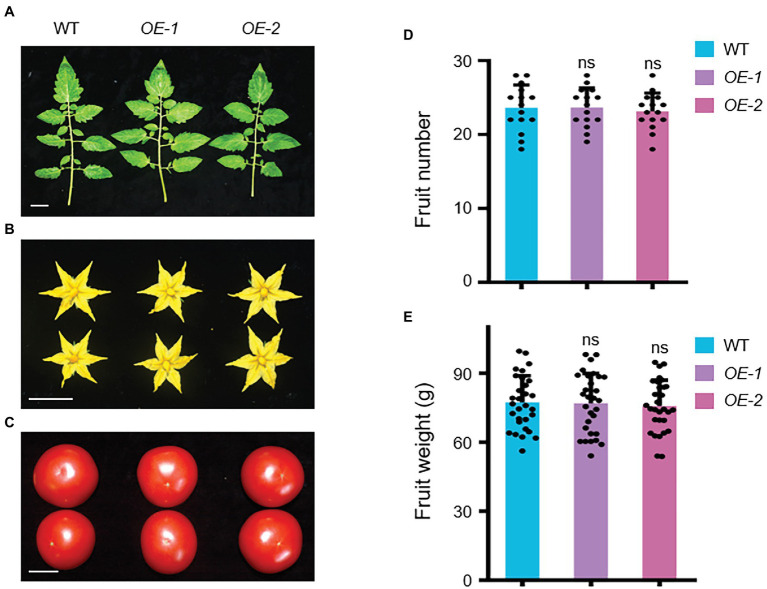
Agronomic Traits of *SlLecRK1*-*OE* Plants. The leaves **(A)**, flowers **(B)**, and fruits **(C)** were observed in two *OE* plants compared with the WT control at the harvest stage. Bars, 3 cm. **(D)** The statistical data for fruit number (*n* = 10 plants). **(E)** The statistical data for fruit weight (*n* = 30 from 10 plants). Different letters denote significant differences by Student’s *t*-test (ns, not significant).

Several agronomic traits were subsequently investigated in *SlLecRK1 OE* plants. We found no visible difference in the fruits, leaves, flowers, and yields between our *OE* and WT plants (Figure 5; Supplementary Figure S1). These observations suggested that the overexpression of *SlLecRK1* does not alter plant growth and development but can improve the resistance of tomato plants to *FORL*; thus, *SlLecRK1* is an ideal candidate for use in future molecular breeding programs.

### Upregulation of *ERF* Genes in the *SlLecRK1-OE1* Lines May Have Contributed to Improve *FORL* Resistance

To clarify the molecular basis for the enhanced resistance of the OE lines, we performed a transcriptome analysis using WT and *OE-1* (*OE*) seedlings without inoculation and at 1 day post-inoculation (dpi) with *FORL* at the stem base; they were designated as WT-0dpi, WT-1dpi, OE-0dpi, and OE-1dpi, respectively. Three biological replicates were prepared for each sample, from which approximately 534.2 million 150-bp paired-end reads were generated (Supplementary Table S2). After discarding low-quality reads, 41.4 to 50.2 million reads were kept for subsequent analysis. More than 85% of reads containing no more than three mismatches were mapped to the reference genome, and more than 80% of the reads showed unique alignments with the samples without *FORL* inoculation, of which the percentage of corresponding reads was much higher than that for the samples after *FORL* inoculation (Supplementary Table S2). Principal component analysis (PCA) demonstrated high reproducibility among the biological replicates (Supplementary Figure S2). Furthermore, PCA revealed that the divergence in the expression profiles of the samples was primarily explained by *FORL* inoculation and that *FORL* inoculation amplified the difference in expression profiles between the *OE* and WT plants (Supplementary Figure S2).

**Figure 6:**
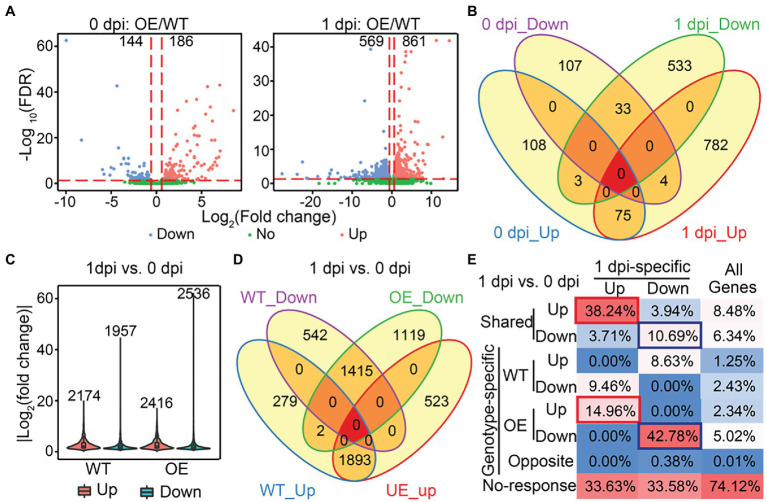
Identification of SlLecRK1-regulated genes during resistance to *FORL* inoculation. **(A)** Volcano plots of the transcriptional changes were generated using edgeR. The log2 of the fold change is shown on the horizontal axis, and the -log10 of the adjusted value of *p* (FDR) is shown on the vertical axis. Red and blue dots represent significantly upregulated and downregulated genes, respectively. Green dots are genes with a non-significant change. The vertical and horizontal red dashed lines indicate the cutoff of the log2-transformed fold change and -log10 of the FDR. Numbers indicate the counts for genes showing upregulation and downregulation. **(B)** A Venn diagram showing the numbers of upregulated and downregulated genes between *OE-1* and WT plants at 0 and 1 dpi. **(C)** The numbers and fold change distributions of DEGs regulated by *FORL* inoculation in WT and *OE-1* plants. **(D)** A Venn diagram showing the comparison of DEGs induced by *FORL* inoculation between WT and *OE-1* plants. **(E)** The categories and percentages of 1dpi-specific upregulated and downregulated genes according to the DEGs caused by *FORL* inoculation in WT and *OE-1* plants. Red and blue boxes indicate type I and type II target genes, respectively. “All genes” indicates genes that were subjected to differential expression analysis.

To identify biological pathways that may be modulated by SlLecRK1 during *FORL* inoculation, we initially compared the transcriptomes of *OE* and WT plants under control conditions (0 dpi: *OE*/WT) and at 1 dpi (1 dpi: *OE*/WT) using a fold change >1.5 and false discovery rate (FDR) < 0.05 as our cutoff values. In total, we identified 186 and 861 upregulated genes and 144 and 569 downregulated genes in the *OE* compared to the WT plants at 0 dpi and 1 dpi, respectively (Figure 6A; Supplementary Dataset S1). Consistent with our PCA results, overexpression of SlLecRK1 resulted in a much greater number of differentially expressed genes (DEGs) at 1 dpi (1430) than under control conditions (330; Figure 6A; Supplementary Dataset S1). Moreover, the number of upregulated genes was higher than that of downregulated genes under both conditions (Figure 6A; Supplementary Dataset S1). Compared with 0 dpi: *OE*/WT comparison, 782 (90.82%) upregulated and 533 (93.67%) downregulated genes were found only in our 1 dpi: *OE*/WT comparison (Figure 6B); these were designated 1dpi-specific genes. Next, we examined whether the 1dpi-specific genes mediate tomato resistance to *FORL* inoculation. First, we evaluated transcriptional changes in response to *FORL* inoculation in WT and *OE* plants; these were designated WT:1dpi/0dpi and OE:1dpi/0dpi, respectively. Using a |log2-transformed fold change| ≥ 2 and FDR < 0.05 as cutoff values, we identified 4,131 DEGs in the WT:1dpi/0dpi comparison, of which 2,174 and 1957 genes were upregulated and downregulated, respectively (Figure 6C; Supplementary Dataset S1). The numbers of DEGs (4952), upregulated genes (2416), and downregulated genes (2536) in the *OE* plants were all higher than those in wild type (Figure 6C; Supplementary Dataset S1). Subsequent comparisons revealed that 87.07% of the activated (1893/2174) and 72.30% of the repressed (1,415/1957) genes in WT plants were also upregulated or downregulated in the *OE* plants (Figure 6D). Collectively, these results suggested that a comparable but more extensive and dynamic transcriptome reprofiling occurred in the *OE* plants following *FORL* inoculation.

Subsequently, we examined the relationships between the SlLecRK1-mediated genes and *FORL*-triggered DEGs to determine the target genes whose transcription was affected by SlLecRK1 during *FORL* inoculation. Interestingly, *FORL*-responsive genes were highly enriched among the 1dpi-specific genes compared with the background (Figure 6E). Among 782 1dpi-specific upregulated genes, 299 (38.24%) and 117 (14.96%) genes, referred to as type I target genes, exhibited shared and *OE*-unique increased expression levels after *FORL* inoculation (Figure 6E; Supplementary Dataset S2), respectively. Meanwhile, 57 (10.69%) and 228 (42.78%) out of 533 1dpi-specific downregulated genes, referred to as type II target genes, exhibited both shared and *OE*-exclusive repression following *FORL* inoculation (Figure 6E; Supplementary Dataset S2), respectively. Considering that SlLecRK1 is a positive regulator of *FORL* resistance, the type I target genes were subjected to further analysis. Unexpectedly, Gene Ontology (GO) analysis revealed that no specific GO terms were significantly enriched among the type I target genes. Still, some transcription factors (TFs) or transcriptional regulators (TRs) might play important regulatory roles in plant resistance to *FORL*. Using the iTAK pipeline (Zheng et al., 2016), we identified and classified 38 putative TF (35) and TR (3) members from 20 families among the type I target genes, including nine genes encoding ethylene (ET)-responsive TFs (ERFs).

**Figure 7:**
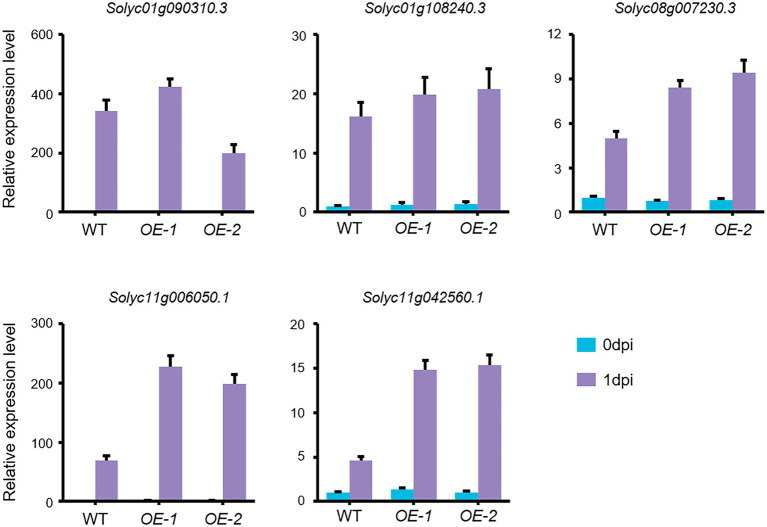
*FORL*-Induced *ERF* Expression in *SlLecRK1*-OE plants. The expression of five *ERF* genes in WT, *OE-1*, and *OE-2* plants before (0 dpi) and after (1 dpi) *FORL* inoculation is shown. Total RNA was used for qRT-PCR analysis. *eEF1α* served as a reference. The WT values at 0 dpi were set to 1. Data are shown as the mean ± SD from three biological repeats.

ERF gene family members have been identified as key mediators of pathogen responses and ET signaling plays important roles in defense responses to necrotrophic pathogens in plants (Spoel and Dong, 2008; Manzo-Valencia et al., 2016). We therefore used qRT-PCR to validate the expression levels of five selected *ERFs*. Consistent with our RNA-Seq data, our qRT-PCR results revealed that the expression levels of the five *ERFs* were substantially elevated in both *OE-1* and *OE-2* plants compared with wild type after *FORL* inoculation (Figure 7). Together, these findings suggest that the upregulation of *ERF* gene expression in response to increased *SlLecRK1* expression contributed to the improved *FORL* resistance of tomato plants.

## Discussion

SlLecRK1, which is mainly expressed in the roots and stem bases of tomato plants, is a membrane-localized RLK. SlLecRK1 belongs to the LecRK subfamily in tomato, and it shows 44% identity to LecRK-I.9/DORN1 in *Arabidopsis*. The L-lectin domain in its extracellular region is predicted to bind monosaccharides and polypeptides, and it is regarded as a potential linker of the plasma membrane to the cell wall, which is the first barrier of plant cells against environmental stimuli (Vaid et al., 2013). Furthermore, SlLecRK1 mRNA and protein are mainly expressed in roots and stems, and their expression levels are increased by *FORL* inoculation. These features render SlLecRK1 able to directly resist *FORL* in soil. Importantly, overexpression of SlLecRK1 does not affect tomato plant growth and development. These data indicate that SlLecRK1 has great potential for use in molecular breeding programs aimed at developing *FORL*-resistant tomato varieties.

SlLecRK1 triggers ET signaling, which mediates defense responses in tomato. ET biosynthesis and signaling are not only modulated by multiple environmental factors such as light, temperature, mechanical pressure, and biotic stress, they also are involved in the regulation of plant growth, development, fruit ripening, and senescence (Cao et al., 2007; Bari and Jones, 2009; Merchante et al., 2013; Wang et al., 2013; Garcia et al., 2015; Larsen, 2015; Huang et al., 2016; Shi et al., 2016; Fei et al., 2019; Seo and Yoon, 2019; Binder, 2020; Husain et al., 2020; Li et al., 2020; Riyazuddin et al., 2020). ERF is a large TF family in plants. ERFs usually bind to the GCC-box *cis*-element GCCGCC, which is frequently present in the promoter region of pathogen-induced genes (Pre et al., 2008; Meng et al., 2013; Catinot et al., 2015). For instance, in *Arabidopsis* ERF6 activates two defense-related genes, *PDF1.1* and *PDF1.2*, *via* an ET-independent pathway in response to infection by the necrotrophic pathogen *B. cinerea* (Meng et al., 2013). Meanwhile, AtERF72 is a positive regulator that mediates resistance to *B. cinerea* by activating the transcription of camalexin-biosynthesis enzyme genes (Li et al., 2021). There are 134 ERFs in tomato, and *SlERF01* and *SlERF2* positively regulate resistance to *Stemphylium lycopersici* (Yang et al., 2020, 2021). *SlERF.A1*, *SlERF.B4*, *SlERF*.*C3*, and *SlERF*.*A3* are required for resistance to *B. cinerea* in tomato (Ouyang et al., 2016). Based on our RNA-Seq and qRT-PCR data, we found that the expression levels of at least five *ERFs* between WT and *SlLecRK1-OE* plants were slightly different before inoculation, whereas the expression levels of these *ERFs* in *SlLecRK1-OE* plants were significantly higher than those in WT plants after inoculation. These results suggest that SlLecRK1 improves *FORL* resistance in tomato by enhancing *ERF* expression. They also explain the phenotypic outcome observed in *SlLecRK1-OE* plants following *FORL* infestation. We also found that SlLecRK1 did not alter *ERF* expression in the absence of *FORL*, while expression was induced by *FORL* infestation. This indicates that the positive effects of SlLecRK1 on *FORL* infestation depend not just on its abundance; it is also likely achieved by another mechanism such as the activation of SlLecRK1 kinase activity by *FORL*. Given these results, we can easily understand why SlLecRK1 overexpression had a reduced impact on tomato plant growth and development. Additionally, SlLecRK1 triggers ET-related gene expression, probably *via* a conserved LecRK-regulated mechanism that has been reported in other plant species under different conditions (Li et al., 2014; Jewell and Tanaka, 2019).

Recently, the *Fusarium* crown and root rot resistance (*Frl*) locus, which confers resistance to FCRR, was mapped to a 900-kb region of chromosome 9 (Devran et al., 2018). Interestingly, *SlLecRK1* is also located in this region. It will be interesting to test whether *SlLecRK1* is *Frl* and to identify the association of *SlLecRK1* with FCRR resistance using natural populations. In summary, we discovered that SlLecRK1 is a specific RLK that regulates the resistance of tomato plants to *FORL*. Additional studies aimed at uncovering the ligands and new component(s) of the SlLecRK1-regulated signaling pathway will aid in clarifying the regulatory mechanism underlying defensive responses to *FORL* and promote tomato improvement.

## Data Availability Statement

The original contributions presented in the study are publicly available. This data can be found at: National Center for Biotechnology Information (NCBI) BioProject database under accession number PRJNA791339.

## Author Contributions

Z-MW and Z-LY: conceptualization and funding acquisition. Z-LY: methodology, data curation, and writing—original draft preparation. Z-LY, Z-JT, J-WZ, and Y-DL: validation. Z-LY, S-WZ, and Z-MW: writing—review and editing. Z-MW: supervision and project administration. All authors contributed to the article and approved the submitted version.

## Funding

This research was funded by Postdoctoral Research Foundation of Hebei Province (B2020003040). Talents Construction Project of Science and Technology Innovation, Hebei Academy of Agriculture and Forestry Sciences (2019-10-10). S&T Program of Hebei (19226342D).

## Conflict of Interest

The authors declare that the research was conducted in the absence of any commercial or financial relationships that could be construed as a potential conflict of interest.

## Publisher’s Note

All claims expressed in this article are solely those of the authors and do not necessarily represent those of their affiliated organizations, or those of the publisher, the editors and the reviewers. Any product that may be evaluated in this article, or claim that may be made by its manufacturer, is not guaranteed or endorsed by the publisher.

## Footnotes

^1^ http://www.bioinformatics.babraham.ac.uk/projects/fastqc/

^2^ https://github.com/najoshi/sickle

^3^ http://www-huber.embl.de/users/anders/HTSeq/doc/overview.html

^4^ https://github.com/js229/Vennerable

^5^ https://imagej.nih.gov/ij/

^6^ https://phytozome-next.jgi.doe.gov/info/Slycopersicum_ITAG4_0
