# Supplementary material for: Overexpression of Lectin Receptor-Like Kinase 1 in Tomato Confers Resistance to Fusarium oxysporum f. sp. Radicis-Lycopersici
Source: Front Plant Sci. 2022 Feb 3;13:836269. doi: 10.3389/fpls.2022.836269 (PMC8850989; doi:10.3389/fpls.2022.836269)
Supplement: Supplementary file 1 [file Table_1.DOCX]

Table S1. List of Primers Used in This Study.

Cloning

| Name | Sequence (5’ to 3’) |
| --- | --- |
| pSlLecRK1 F | CCCAAGCTTCCTCTCTCACTCCATCTCTCG |
| cSlLecRK1 R | GCGGATCCCCTAGGCAACTCATCAGTAAAT |
| ICD F | CACCTTAGTTAGGAGATTCAAGAATGC |
| ICD R | CCTAGGCAACTCATCAGTAAAT |

qPCR

| Name | Sequence (5’ to 3’) |
| --- | --- |
| Solyc05g053010 F | GGGAATGTGTTGTTAGATTCCG |
| Solyc05g053010 R | AAAACATCTGAACTTGTCGTGG |
| Solyc07g065610 F | TCTTTGTTCCGAAAGTGAAACC |
| Solyc07g065610 R | CTGATTTCGAGCTCATGTTCTG |
| Solyc09g005000 F | GAGCTCTCATTACATCTTGGGT |
| Solyc09g005000 R | CTATATCGGAACCTGTGAGGAC |
| Solyc09g007510 F | AGTACAGAAAAACAGCAGCAAG |
| Solyc09g007510 R | GAAGCCTTCACATGCTTGAATT |
| Solyc09g011060 F | GGACTTCAGGGGTCTTTGGC |
| Solyc09g011060 R | AACCTGCTGTGTGAATTGCTAC |
| Solyc09g011070 F | TGAGCTTCTTGGGTTTGGGG |
| Solyc09g011070 R | ACCACGACGTCTACACCAAC |
| Solyc09g011990 F | CGATGGTCTTTTGCTGTTAACA |
| Solyc09g011990 R | TAATCAGACCTTATGGCGAACA |
| Solyc09g012000 F | TGTTCGCGATAAGGTCTGATTA |
| Solyc09g012000 R | ATTCATATCACCAAAATCCGCG |
| Solyc10g084250 F | AGGGTTCAATGCAAGATTAGGA |
| Solyc10g084250 R | TAACAAGTACTCTGGAGCCAAG |
| Solyc10g084860 F | CATGAAGGCTATGAACAAGTCG |
| Solyc10g084860 R | CTTTAGTCGTGGACGGATTAGA |
| eEF1α/Solyc06g069020F | CCACCTCGAGATCCTAATGG |
| eEF1α/Solyc06g069020 R | ACCCTCACGTATGCTTCCAG |
| Solyc11g006050 F | GGTTTGGTTAGGGACATTTGAC |
| Solyc11g006050 R | TCAATCTCCATCAACGATCGAT |
| Solyc01g090310 F | TTTTCCTCATTTAATCGGCTCG |
| Solyc01g090310 R | TGGTGTTCCATTTTCACATGAC |
| Solyc08g007230 F | TTTTCCGTTGAGGGTTAATTCG |
| Solyc08g007230 R | GATGACGAAGAAAAACTCTCCG |
| Solyc11g042560 F | AGTAAGGAAGAGAAAATGGGGG |
| Solyc11g042560 R | AGGGTTTTGGGAATGTATGGAT |
| Solyc01g108240 F | TAGTACAGCTACAGTCGCAATC |
| Solyc01g108240 R | GCTTTTCGAGGATCACGAATTT |
| OMP1049 | TGCGATTTGGACGAGATATGTG |
| OMP1050 | ATTTGCCTACCCTGTACCTACC |

Site-directed mutagenesis

| Name | Sequence (5’ to 3’) |
| --- | --- |
| SlLecRK1 K-A F | TAGCTGTGGCGCGTATTTCACATGAATCTAAACAAGG |
| SlLecRK1 K-A R | GAAATACGCGCCACAGCTATTTCCATATTCGAACTTTG |
