# Supplementary material for: Overexpression of Lectin Receptor-Like Kinase 1 in Tomato Confers Resistance to Fusarium oxysporum f. sp. Radicis-Lycopersici
Source: Front Plant Sci. 2022 Feb 3;13:836269. doi: 10.3389/fpls.2022.836269 (PMC8850989; doi:10.3389/fpls.2022.836269)
Supplement: Supplementary file 5 [file Data_Sheet_1.PDF]

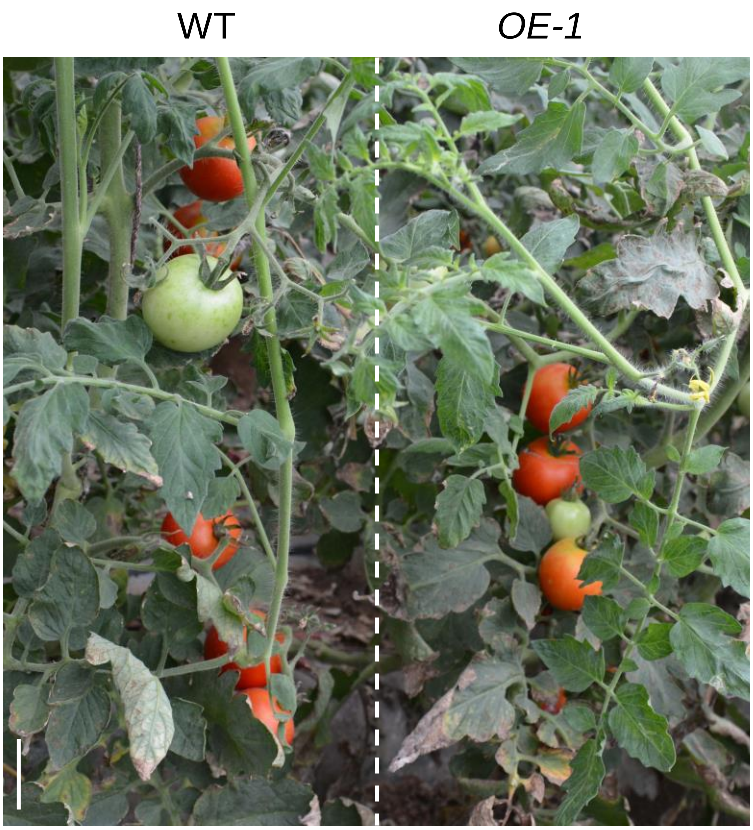

Figure S1. WT and *OE-1* Plants at the Harvest Stage.
Phenotypes of 100-day-old WT and *OE-1* tomato plants. Bar, 5 cm.

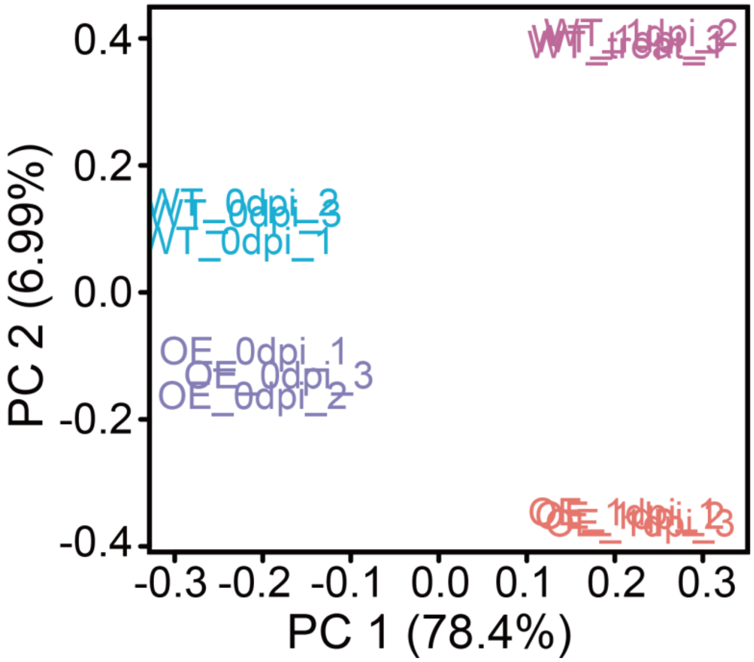

Figure S2. Results of the PCA of All Sequenced Samples.

PCA of all sequenced samples. WT-0dpi and OE-0dpi (*OE-1*) indicate 10-day-old WT and *OE* seedlings without *FORL* inoculation. WT-1dpi and OE-1dpi indicate WT and *OE* seedlings at 1 day after *FORL* inoculation.
